# Research in Mindfulness Interventions for Patients With Fibromyalgia: A Critical Review

**DOI:** 10.3389/fnint.2022.920271

**Published:** 2022-07-28

**Authors:** Salomé Leça, Isaura Tavares

**Affiliations:** ^1^Unit of Experimental Biology, Department of Biomedicine, Faculty of Medicine, University of Porto, Porto, Portugal; ^2^Institute of Molecular and Cell Biology, University of Porto, Porto, Portugal; ^3^I3S–Institute of Investigation and Innovation in Health, University of Porto, Porto, Portugal

**Keywords:** chronic pain, mind-body interventions, meditation, emotional-regulation, selfregulation, clinical trials, oriented review

## Abstract

Fibromyalgia is one of the most common causes of widespread chronic pain. It has a huge impact on the quality of life, namely because it appears earlier in life than most of the chronic pain conditions. Furthermore, emotional-cognitive distress factors, such as depression and anxiety, are a common feature in patients with fibromyalgia. The neurobiological mechanisms underlying fibromyalgia remain mostly unknown. Among non-pharmacological treatments, cognitive-behavioral therapy has been used during the last decade, namely with the enrolment of patients in programs of mindfulness-based stress reduction (MBSR) and in mindfulness-based interventions (MBI). We critically analyzed the literature to search for scientific evidence for the use of MBI in fibromyalgia. The studies were evaluated as to several outcomes of fibromyalgia improvement along with aspects of the study design which are currently considered relevant for research in mindfulness. We conclude that despite the sparsity of well-structured longitudinal studies, there are some promising results showing that the MBI are effective in reducing the negative aspects of the disease. Future design of studies using MBI in fibromyalgia management should be critically discussed. The importance of active controls, evaluation of sustained effects along with investigation of the subserving neurobiological mechanisms and detailed reports of possible adverse effects should be considered.

## Introduction

Fibromyalgia is a complex and debilitating disease. Its main symptoms are chronic widespread pain, sleep problems, physical exhaustion, and cognitive difficulties (Hauser et al., 2015). Patients suffering from this condition also have a high variety of somatic symptoms, psychological distress and present a higher prevalence of depression and anxiety-related disorders (Fietta et al., 2007).

There is no gold standard test for fibromyalgia diagnosis. The more recent criteria of the American College of Rheumatology (ACR) to identify fibromyalgia postulate that the diagnosis in adults should meet the following criteria (Wolfe et al., 2016):

1. Generalized pain, defined as pain in at least 4 of 5 regions;
2. Symptoms present at a similar level for at least 3 months;
3. Widespread pain index (WPI) ≥ 7 and symptom severity scale (SSS) score ≥ 5 OR WPI of 4–6 and SSS score ≥ 9;
4. A diagnosis of fibromyalgia is valid irrespective of other diagnoses. A diagnosis of fibromyalgia does not exclude the presence of other clinically important illnesses.

Most studies show that fibromyalgia prevalence is approximately 2% in the general population, having a higher impact in women. However, one systematic review in the world using meta-analyses (Heidari et al., 2017) showed that this general population prevalence is much lower than the prevalence of fibromyalgia among patients with specific disorders, such as irritable bowel syndrome (12.9%), hemodialysis (6.3%), and type 2 diabetes mellitus (14.80%).

The pathophysiology of the disease is under active research but remains unknown. Two main neurobiological mechanisms have been proposed. The first is an alteration of the immune-inflammatory connection which may lead to sensitization of the peripheral and central nervous system. Patients with fibromyalgia present an imbalance of pro-inflammatory and anti-inflammatory cytokines, with an increase in the former and a decrease in the later (Uceyler et al., 2011; Rodriguez-Pinto et al., 2014; Littlejohn, 2015; Backryd et al., 2017). The second mechanism derives from the strong link between pain complaints and emotional/cognitive distress consistently reported in patients with fibromyalgia and proposes that peripheral causes are less relevant than central consequences. This so-called “brain hypotheses” (Ceko et al., 2013; Hubbard et al., 2020) considers that fibromyalgia is a central sensitivity syndrome characterized by sensitization of the somatosensory system, a condition common to chronic pain situations, in which a signal amplification occurs during the transmission of nociceptive input, which leads to exacerbation of pain perception (Vierck, 2006). Besides this somatic sensitization, fibromyalgia patients also present “cognitive-emotional sensitization,” a cognitive bias toward the negative events accompanied with perseverative negative thoughts, rumination and catastrophizing (Brosschot, 2002). A recent imaging study proposed that fibromyalgia patients with more severe pathology in the peripheral nervous system presents higher alterations in morphology, structural and functional connectivity at the encephalon, which allows to connect the peripheral and brain mechanism reviewed above (Aster et al., 2022).

Pain is modulated from the brain, and several chronic pain conditions have been ascribed to deficient top-down pain modulation that is unable to block the transmission of input from the spinal cord or may even increase neuronal transmission through the somatosensory system (Heinricher et al., 2009). A meta-analysis of neuroimaging studies showed that in fibromyalgia there is an increased neuronal activation in pain processing areas, such as the posterior insula and secondary somatosensory cortices, along with altered functional connectivity in brain areas included in the pain matrix related to central sensitization (O’Brien et al., 2018). Also, in fibromyalgia patients, increased connectivity was detected between the insula (a pronociceptive region) and the “default mode network,” which is active when the brain is at rest (Napadow et al., 2010; Jensen et al., 2012). The opposite, decreased connectivity, occurs between brain areas involved in pain inhibition (Napadow et al., 2010; Jensen et al., 2012). These results are in accordance with other studies and indicate impaired top-down pain modulation during fibromyalgia.

Because of its multifactorial and poorly understood etiopathogenesis, fibromyalgia has no curative treatment. The current modalities aim to target the main symptoms of the disease and improve the quality of life (QoL). However, because of the high variability in patient-to-patient symptoms’ predominance and severity, the approach needs to be empirical, individualized, and based upon a strong therapeutic alliance between clinician and patient with realistic treatment goals.

The European League Against Rheumatism (EULAR) revised recommendations for managing fibromyalgia, separates the specific recommendations in non-pharmacological and pharmacological therapies (Macfarlane et al., 2017). It recommends starting the treatment with non-pharmacological modalities, namely, physical exercise, the only “strong for” recommendation, with effects on pain, physical function and wellbeing. Additional non-pharmacological therapies can be combined, such as meditative movement therapies (qigong, yoga, tai chi → effects on sleep, fatigue, and QoL), mindfulness-based stress reduction (pain and QoL), acupuncture (pain and fatigue), and hydrotherapy (pain and QoL).

If therapeutic failure within this first step, an additional individualized treatment is recommended. For those with mood disorders or unhelpful coping strategies, cognitive behavioral therapy should be considered. Patients with severe pain or severe sleep disturbance should be considered for pharmacotherapies, specifically duloxetine, pregabalin and tramadol for pain and amitriptyline, cyclobenzaprine and pregabalin for sleep (Macfarlane et al., 2017). However, some “transatlantic” differences emerged namely as to the use of duloxetine and pregabalin namely given the small effect sizes and the associated adverse effects (Briley, 2010). Ultimately, multimodal rehabilitation programs will be necessary for patients with severe disability, with stronger improvements than individual therapies alone.

## Mind-Body Interventions: Mindfulness

The use of mind-body interventions has increased dramatically in the last two decades in Western countries with the huge challenge of conciliating evidence-based medicine with traditions and practices that are common for centuries in the East.

Departing from the Buddhist philosophy, mindfulness has reached the Western mainly by the structured programs of mindfulness-based stress reduction (MBSR) implemented in 1979 by Kabat-Zinn (1982). Departing from MBSR programs, other mindfulness approaches such as mindfulness-based cognitive therapy (MBCT) and mindful-self compassion (MSC) have been applied for the treatment of clinical disorders such as anxiety, depression and stress along with diseases with a huge psychological burden and impact in the QoL, such as chronic pain (Khoury et al., 2013; Goyal et al., 2014; Creswell, 2017).

These programs typically last 8 weeks and its primary theoretical premise is that, by practicing mindfulness, individuals will become less reactive to unpleasant phenomena and more contemplative and reflective, leading to increased self-awareness and self-emotional regulation (Creswell, 2017). Mindfulness is an active and intentional practice that may lead to a mental condition characterized by non-judgmental awareness of the experience in the present moment, including one’s sensations, thoughts, bodily states, consciousness, and the environment, while encouraging openness, curiosity, and acceptance (Bishop et al., 2004).

Mindfulness involves two components: self-regulation of attention and orientation toward the present moment with curiosity, openness, and acceptance (Bishop et al., 2004). Although they are not mindfulness-structured programs, mindfulness is present in other evidence-based cognitive-behavioral therapies, such as dialectical behavior therapy (DBT) and acceptance and commitment therapy (ACT). Despite the exponential increase in the number of published studies with the application of Mindfulness Based Interventions (MBI) in the last decades, a considerable bulk of research comes from cross-sectional studies, waitlist-controlled trials, and other methodological shortcomings that reduce the strength of the conclusions. A continuous monitoring of the quality of research using MBI has been proposed by several researchers.

## Mind-Body Approaches in Fibromyalgia: Mindfulness Based Interventions

There are at least four major challenges in fibromyalgia treatment. The first derives from the fact that fibromyalgia is hard to diagnose. After lombalgia and osteoarthritis, fibromyalgia is usually considered the third most prevalent musculoskeletal pain-associated condition, but it remains underdiagnosed and is considered a “mysterious syndrome” (Sarzi-Puttini et al., 2020). The second challenge derives from its unclear etiopathogenesis which precludes a fully based mechanistic treatment (Perrot, 2019). The third and fourth challenges are common to some pain conditions, namely the lack of standard biomarkers (signatures) and interindividual variability of complaints and responses to treatment. Both challenges may be due to the concept of pain.

The International Association for the Study of Pain (IASP) defines pain as “an unpleasant sensory and emotional experience associated with, or resembling that associated with, actual or potential tissue damage” (Raja et al., 2020). Pain is not only a sensory experience, and its cognitive and emotional components demand a multifactorial approach which is far beyond pharmacological management and frequently requires psychological interventions aimed to promote coping strategies and emotional regulation. These psychological approaches represent a possibility for individually tailored interventions, where pharmacological treatments are insufficient.

Also demonstrating the importance of psychological factors, expectations are important in clinical management of fibromyalgia. One systematic review and meta-analysis of randomized controlled trials showed that the size effect of placebo for pain relief was clinically moderate (0.53, 95% CI 0.48–0.57). The same review concludes that placebo treatment was shown to also reduce fatigue and improve non-restorative sleep and increase the QoL mainly in women and initial phases of the disease (Chen et al., 2017).

Mindfulness may be defined as “the awareness that arises through paying attention, on purpose, in the present moment, non-judgmentally” (Kabat-Zinn, 1982). By promoting acceptance and fostering emotional self-regulation, MBI may theoretically be suitable for managing distress events in patients with fibromyalgia. From the multiple studies using MBI in those patients, the majority have used MBSR programs.

Based on the critical perspectives and concerns about research in mindfulness (Davidson and Kaszniak, 2015; Van Dam et al., 2018), we conducted a critical review of the literature with two aims 1. Analyze the evidences of benefits of MBI in patients with fibromyalgia and 2. Establish if the available papers used the directives of research in this field, namely the existence of active controls, reports of previous experience of participants in mind-body approaches, evaluation of expectations of the participants and meditation experience of the teachers. The purpose of this critical review was not to perform a systematic review of the literature since there are several studies that evaluated the effects of MBI interventions for fibromyalgia (Lauche et al., 2013; Haugmark et al., 2019; Khoo et al., 2019; Pei et al., 2021).

We conducted a research on database PubMed, using the terms “(mindfulness or meditation) and fibromyalgia”. Our inclusion criteria were:

1. Randomized controlled trials and non-randomized controlled trials.
2. Studies of patients diagnosed with fibromyalgia (no other chronic pain conditions).
3. Studies that compared well-structured MBI programs with any active treatment.
4. Studies that assessed at least one patient-centered outcome, with no restrictions applied.

From the 160 initial results, we first removed all duplicates. Then, titles and abstracts were individually screened for potentially eligible studies. The remaining 30 studies passed through a full text assessment and 11 were excluded for not being controlled trials, seven for not having an active intervention for comparison, four for not having well-structured MBI programs within the intervention group and one for not assessing patient-centered outcomes. This resulted in seven eligible studies, which are listed in Table 1, in chronological order of publication.

**TABLE 1:** Overview of included studies’characteristics and main findings.

Author, year, (reference)	Participants (N, age, gender, drop-out rate)	Co-occurring therapies	Intervention	Comparison (control)	Outcome measures	Measurement time point	Results		Self-reported study limitations
								
							Short-term	Long-term	
Grossman et al. (2007)	58 fibromyalgia patients Mean age MBSR group: 54.40 ± 8.30 years Control group: 48.80 ± 9.10 years Gender: female only Drop-out: 10,30%	Not detailed	MBSR (n= 39) Kabat-Zinn adapted program 8 weeks total One 2.5h weekly group- session + all-day retreat (7h) + 45-min daily self- practice	Social support, relaxation, stretching. (n=13) 8 weeks total One 2.5h weekly session + 45 min daily self-practice	1) Pain severity (VAS) 2) Pain perception (PPS) 3) Pain regulation (l PR) 4) Quality of life (PLC) 5) Anxiety and Depression (HADS) 6) Physical symptoms	Baseline Post-treatment 3 years follow-up (only for MBSR group)	No between-groups comparisons made. MBSR: Control group: 1) Sign, improvement 1) No sign.imp. 2) Sign, improvement 2) No sign.imp. 3) Sign, improvement 3) No sign.imp. 4) Sign, improvement 4) No sign.imp. 5) Sign, improvement 5) No sign.imp. 6) Sign, improvement 6) No sign.imp.	MBSR only (n=26): 1) Sign. improvement 2) No sign. improvement 3) Sign. improvement 4) Sign. improvement 5) Sign. improvement 6) Sign. improvement	Not randomized Female patients only Small control group Baseline differences between experimental groups No medical utilization Assessed Follow-up did not include the control group
Schmidt et al. (2011)	177 fibromyalgia patients Mean age: 52.50 ± 9.60 years Gender: female only Drop-out: 5,10%	Usual care Not detailed	MBSR (*n* = 53) Kabat-Zinn based program 8 weeks total One 2.5h weekly group- session + all-day retreat + 45-60 min daily self-practice.	Wait-list (n=59) No-treatment Active intervention (N=56) Support, education, relaxation, stretching. 8 weeks One 2.5 h weekly session + 45–60 min daily self practice	1) Pain perception (PPS) 2) Quality of life (PLC, FIQ) 3) Quality of sleep (PSQI) 4) Depression (CES-D) 5) Anxiety (STAI) 6) Physical symptoms (GCQ)	Baseline Post-treatment 2 months follow- up	1) No sign, group differences 2) No sign, group differences 3) No sign, group differences 4) No sign, group differences 5) No sign, group differences	1) No sign. group differences 2) No sign. group differences 3) No sign. group differences 4) No sign. Group differences 5) No sign. Group differences	Excessive patient burden Recruitment and assignment Treatment fidelity
Davis and Zautra (2013)	79 fibromyalgia patients Mean age: 46.14 years Gender: 77 female (97,50%) 3 male (2,50%) Drop-out: 0,00%	Not detailed	MSER (n=39) Online. Up to 6 weeks duration. 12 modules, self-paced. One Adobe Presentation lasting 15 min for each module. + Encouragement to use the skills discussed in each module. + Audio-recording of 1 mindfulness meditation relevant for each module plus encouragement to access it daily + Daily reports	Health tips (n=40) Information regarding health- promoting behaviors. Online. Up to 6 weeks duration. 12 modules, self-paced. One Adobe Presentation lasting 15 min for each module + Daily reports	1) Pain 2) Pain Coping Efficacy 3) Positive and Negative Affect (PANAS) 4) Perceived Social Relations (Social activity engagement, Loneliness, Family stress, Family enjoyment)	Baseline Post-treatment	1) No sign, group differences 2) Sign, group differences favoring MBI 3) PA: Sign, group differences favoring MBI NA: No sign, group differences 4) Sign, group differences favoring MBI		No confirmation of Fibromyalgia diagnosis Untested generalizability of findings Self-reported outcome measures No evaluation of treatment effects mechanisms No follow-up Preliminary findings
Grossman et al. (2017)	168 fibromyalgia patients Mean age: 52.50 ± 9.60 years Gender: female only Drop-out: 16,70%	Usual care 46% taking antidepressants (no group differences)	MBSR Kabat-Zinn based program 8 weeks total One 2.5h weekly group-session + all-day retreat + 45-60 min daily self-practice.	Wait-list No-treatment Active intervention (N= 59) Support, education, relaxation, stretching. 8 weeks One 2.5h weekly session + 45-60 min daily self-practice	1) Cardiac autonomic regulation (HR, RSA) 2) Respiratory function (Fb, Vt, V) 3) Physical activity (accelerometer) 4) Association between 1),2),3) and Patient-reported clinical improvement (PPS, PLC, FIQ, PSIQI, CES-D, ST Al, GCQ)	Baseline Post-treatment 2 months follow-up	1) No sign, group differences 2) No sign, group differences 3) No sign, group differences 4) No sign, association despite sign. Patient- Reported clinical improvement	1) No sign, group differences 2) No sign, group differences 3) No sign, group differences 4) No sign, association	Small period of monitoring Excessive patient burden High drop-out rate Co-occurring psychopharmacologic al medication
Van Gordon et al. (2017)	148 fibromyalgia patients Mean age MAT group: 46.41 ± 9.06 years Control group: 47.34 ± 9.83 Gender 123 female (83,10%) 25 male (16,90%) Drop-out: 28,40%	Not detailed	MAT (n=54) 8 weeks total One 2h group weekly group- session (with 5- min break) + 50-min one-to-one support sessions + daily self-practice (dynamic and individualized routine)	CGBT (n=52) Education- focused 8 weeks total One 2h group weekly group-session (with 5- min break) + 50-min one-to- one support sessions + at-home practice	1) Functional Impact (FIQR) 2) Pain perception (SF-MPQ) 3) Sleep quality (PSIQI) 4) Psychological distress (DASS) 5) Non-attachment (NAS) 6) Civic engagement	Baseline Post-treatment 6 months followup	1) Sign, group differences favoring MAT 2) Sign, group differences favoring MAT 3) Sign, group differences favoring MAT 4) Sign, group differences favoring MAT 5) Sign, group differences favoring MAT 6) Sign, group differences favoring MAT	1) Sign, group differences favoring MAT 2) Sign, group differences favoring MAT 3) Sign, group differences favoring MAT 4) Sign, group differences favoring MAT 5) Sign, group differences favoring MAT 6) Sign, group differences favoring MAT	Self-referring participants Reliance on selfreport measures Only three-time points assessments “Popularity Effect’ phenomenon
Cejudo et al. (2019)	117 fibromyalgia patients Mean age 47.59 ± 5.93 years Gender: female only Drop-out: 11,10%	Pharmacological treatment for pain Not detailed	MBI (n= 53) MBSR-adapted: 20 weeks total One 1 h weekly group- session + daily self-practice (audio-guide support + 5 min body scan + 15 min attention focused on breathing)	Psychoeducation (n= 51) Information on common symptoms and advice on self- care	1) Subjective wellbeing (SWLS, PANAS) 2) Trait emotional intelligence (TEIQue- SF) 3) Mental health (MH5) 4) Resilience (ER-14)	Baseline Post-treatment 6 months followup	1) SWLS: Sign, group differences favoring MBI PANAS:Sign. group differences favoring MBI in PA (not in NA) 2) No sign, group differences 3) Sign, group differences favoring MBI 4) Sign, group differences favoring MBI	1) SWLS: Sign, group differences favoring MBI PANAS: Sign, group differences favoring MBI in PA (not in NA) 2) Sign, group differences favoring MBI 3) Sign, group differences favoring MBI 4) Sign, group differences favoring MBI	Sample composed with female only Not objective measures (self-report only) Different MBI structure from other studies
Pérez-Aranda et al. (2019b)	225 fibromyalgia patients Mean age: 52.50 ± 9.60 years Gender: 221 female -98.20% 4 male (1,80%) Drop-out 23,10%	T reatment-as- usual (TAU) Anxiolytics, antidepressant s, opioids, antiinflammatories, and analgesics. (No group differences)	MBSR (n=58) University of Massachusetts Medical School (USA) MBSR protocol: 8 weeks total One 2h weekly group- session + optional 6h retreat + 45 min daily selfpractice.	TAU alone (n=55) No-treatment FibroQoL (n=60) Psychoeducati on, selfhypnosis. 8 weeks One 2h weekly session + 45-60 min daily selfpractice	1) Functional impact (FIQR) 2) “Fibromyalginess” (FSDC) 3) Anxiety and Depression (HADS) 4) Pain catastrophizing (PCS) 5) Perceived stress (PSS-10) 6) Cognitive disfunction (MISCI)	Baseline Post-treatment 12 months follow-up	1) Sign, group differences favoring MBSR over FibroQoL and TAU. 2) Sign, group differences favoring MBSR over TAU (not FibroQoL) 3) Sign, group differences favoring MBSR over TAU and FibroQoL 4) Sign, group differences favoring MBSR over TAU and FibroQoL 5) Sign, group differences favoring MBSR over TAU and FibroQoL 6) Sign, group differences favoring MBSR over TAU and FibroQoL	1) Sign, group differences favoring MBSR over TAU (not FibroQoL). 2) Sign, group differences favoring MBSR over TAU and FibroQoL. 3) Sign, group differences favoring MBSR over TAU (not FibroQoL). 4) Sign, group differences favoring MBSR over TAU and FibroQoL. 5) Sign, group differences favoring MBSR over TAU (not FibroQoL). 6) Sign, group differences favoring MBSR over TAU (not FibroQoL).	Practice frequencydependent outcomes Randomization issues Low follow-up rates (65%) MBSR and FibroQoL differences in therapy time and patient expectations Not optimal adherence to intervention protocols High-time demanding training

*CES-D, Center for epidemiological studies; CGBT, Cognitive behavioral theory for groups; DASS, Depression, Anxiety, and Stress Scale; ER-14, Resilience Scale; Fb, Breathing frequency; FIQ, Fibromyalgia Impact Questionnaire; FIQR, Revised Fibromyalgia Impact Questionnaire; FSDC, Fibromyalgia Survey Diagnostic Criteria; GCQ, Giessen Complaint Questionnaire; HADS, Hospital Anxiety and Depression Scale; HR, Heart Rate; IPR, Inventory of Pain Regulation; MAT, Meditation awareness training; MBSR, Mindfulness-based stress reduction; MH-5, Mental Health Questionnaire; MISCI, Multidimensional Inventory of Subjective Cognitive Impairment; MS ER, Mindful socioemotional regulation; NAS, N on-Attachment Scale; PANAS, Positive and Negative Affection Scale (division in to PA, Positive Affection, and NA, Negative Affection, subscales); NRS-101, 101-point Numerical Rating Scale; PCS, Pain Catastrophizing Scale; PLC, Quality of life Profile for the Chronically III; PPS, Pain Perception Scale; PSQI, Pittsburgh Sleep Quality Index; PSS-10, Perceived Stress Scale; RSA, Respiratory sinus arrhythmia; SF-MPQ, Short-form McGill Pain Questionnaire; STAI, State-Trait-Anxiety-Inventory; SWLS, Satisfaction with Life Scale; TEIQue-SF, Trait Emotional Intelligence Questionnaire Short FormjV, Ventilation; VAS, Visual Analog Scale; V_t,_ Tidal volume.*

Table 1 summaries the results regarding the evidences of benefits of MBI in patients with fibromyalgia (our first aim). We clustered the outcomes as being related to either Fibromyalgia Functional Impact and Symptomatology, Pain, Mental Health, or QoL. We highlight positive findings and outcomes measured through a validated scale. Two studies (Van Gordon et al., 2017; Pérez-Aranda et al., 2019b) assessed Fibromyalgia Functional Impact and Symptomatology-related outcomes (FIQR and FSDC) in which effect sizes in the medium-large range were reported favoring the mindfulness-based intervention (Cohen’s d from 0.35 to 0.86). Three studies (Grossman et al., 2007; Van Gordon et al., 2017; Pérez-Aranda et al., 2019b) specifically assessed Pain-related outcomes, either its objective (pain severity) or subjective (pain perception, regulation, and catastrophizing) experience, with medium-large size effects reported in MBI over control group (Cohen’s d from 0.34 to 1.10). Mental Health-related outcomes, such as anxiety and depression (HADS), mental health (MH5), psychological distress (DASS) and perceived stress (PSS-10) were measured by four studies, showing small-medium effect sizes favoring MBI (Cohen’s d from 0.39 to 0.77; Eta squared h2 0.022) (Grossman et al., 2007; Van Gordon et al., 2017; Cejudo et al., 2019; Pérez-Aranda et al., 2019b). Quality of life-related outcomes included subjective well-being (SWLS), positive and negative affect (PANAS), resilience (ER-14), Sleep quality (PSIQI), non-attachment (NAS), and cognitive dysfunction (MISCI). Effect sizes in the small-big range were reported favoring the MBI (Cohen’s d from 0.52 to 1.12; Eta squared h2 from 0.015 to 0.143) (Grossman et al., 2007; Davis and Zautra, 2013; Van Gordon et al., 2017; Cejudo et al., 2019; Pérez-Aranda et al., 2019b). In conclusion, and regarding our first aim, the global evaluation of the results in Table 1 shows that there is some evidence that MBI are effective in reducing several fibromyalgia outcomes.

As to the second aim of this manuscript namely to analyze if the current literature considered the recommendations for research in Mindfulness (Davidson and Kaszniak, 2015; Van Dam et al., 2018) several conclusions can be outlined. The overall analysis of the published studies shows that only 4.38% had inclusion criteria such as active control groups and randomized controlled trials which lead us to include only seven studies in this critical review. Furthermore, none of the considered studies for analysis reported information which is considered relevant in mindfulness research (Davidson and Kaszniak, 2015; Van Dam et al., 2018), namely detailed the previous mindfulness experience of the enrolled participants and the participants’ interests for medical approaches only or for integrative ones as well. Regarding the teachers’ experience (e.g., “number and type of retreats attended,” “blindness to the research hypothesis”, “conflicts of interest”), the information is scarce or even nil. The type of meditation practiced by the teachers was also not referred. The occurrence of adverse or unpleasant effects of MBI, an issue to consider (Britton et al., 2021), was only reported by Pérez-Aranda et al. (2019b).

## Discussion and Future Perspectives

Several systematic reviews and meta-analysis evaluated the evidences for the use of MBI in fibromyalgia (Lauche et al., 2013; Haugmark et al., 2019; Khoo et al., 2019; Pei et al., 2021).Therefore, the current study did not intend to perform a systematic review but rather a critical analysis of some of the available literature, considering the outcomes of the studies but taking into account the alerts recently raised by researchers in Mindfulness (Davidson and Kaszniak, 2015; Van Dam et al., 2018). The analyzed studies used a wide diversity of outcomes and showed moderate evidence for the use of MBI in fibromyalgia. Considering the limitations of the current analysis, this critical review is in line with the results of the systematic reviews in this field (Lauche et al., 2013; Haugmark et al., 2019; Khoo et al., 2019; Pei et al., 2021).

As to the critical analysis of the methodology used in the studies and taking into account some of the current recommendations regarding research in Mindfulness (Davidson and Kaszniak, 2015; Van Dam et al., 2018), a relevant issue is the inclusion of active controls. The inclusion of active controls is important in what concerns study design namely because it helps to solve the question of double-blinding (Davidson and Kaszniak, 2015). A recent study analyzed the effects of including a validated well-matched active control group with a large sample size and defined in a randomized manner in the effects of MBSR in brain structure (Kral et al., 2022). This study failed to confirm previous results showing neuroplastic changes induced by MBSR groups in comparison to active controls, which reinforces the importance of defining the best control groups for each MBI intervention which may probably require both active and waiting list groups (Kral et al., 2022). As to fibromyalgia, it was never evaluated the importance of defining control groups in MBI research. The complexity which may be introduced by not defining in detail what the active control group is enrolled in, namely if there is a validated and matched structured program or if the waiting list participants spontaneously changed their activities during the participation in the study. As to the studies excluded of the current analysis due to the lack of a control group (Kaplan et al., 1993; Sephton et al., 2007; Amutio et al., 2014, 2018; Cash et al., 2015; Andres-Rodriguez et al., 2019) it should be noted that they were also excluded because of other factors namely because in some of them the interventions could not be considered a MBI or they were just evaluating Mindfulness scores without intervention. Since we were not performing a systematic review of the literature, we consider that the exclusion of those studies from Table 1 is not relevant.

Real life adaptation may be considered in MBI research for fibromyalgia, in agreement to other authors (Mantzios and Giannou, 2019). High drop-out rates and/or low adherence to total completion of intervention is a common feature in the analyzed studies, which was also reported in MBI for health conditions other than fibromyalgia (Zhou et al., 2020). Multiple causes can be appointed to this situation. We first highlight that three of the analyzed studies using MBSR programs as intervention reported limitations such as excessive patient burden (Schmidt et al., 2011; Grossman et al., 2017), no optimal adherence to intervention and high-time demanding training (Pérez-Aranda et al., 2019b). The basic program requires 45 min per day of formal home practice, plus one weekly 2–2.5 h group session and one all-day retreat over the course of 8 weeks. A high and condensed time requirement approach like this may limit the intervention efficiency, considering that fibromyalgia patients will encounter resistance in adapting it to their real life routine and own obstacles—in fact, such a schedule is challenging for anyone to accomplish. There are possible solutions to overcome the problem, such as, providing (1) MBI programs with longer intervention time intervals but with less week load, with shorter daily practices (Cejudo et al., 2019), (2) MBI online-adapted programs, potentiating patient-freedom in time-scheduling their own self-paced sessions (Davis and Zautra, 2013), and (3) an enhancement of group-support sessions to improve adherence. The value of these adaptations may be considered inasmuch that several studies showed that decreasing the duration of the intervention does not preclude efficacy in pain responses (Zeidan et al., 2010). Future research considering the need of MBI programs adaptation to real-life routine, could provide more efficient results among fibromyalgia patients. This may also be important to the continuation of the practice of mindfulness after completion of the interventions which is a problem that can impair long term effects of MBI.

Also regarding adherence to MBI, patient motivation for the intervention is frequently under evaluated. Two of the analyzed studies (Grossman et al., 2007; Schmidt et al., 2011) are from the same research group, and the former is a forerunner study of the latter. Despite sharing similar design, intervention groups (including actual MBSR instructors) and outcome variables, they show different results. Possible reasons for the discrepancies could be the recruitment and assignment of patients to intervention, according to their motivations (Schmidt et al., 2011). While study 27 was based upon patients’ preferences, who were allowed to actively choose the MBSR intervention, study 26 was a randomized assignment. Despite being considered a study with better design quality, due to its randomization, it may diminish the effect that patients’ preference, and consequent motivation for intervention-adherence has on enhancing the effect of the treatment itself. Even if patient motivations are not considered in allocation of the patients to the interventions, it is important to evaluate these parameters for a better analysis of the intervention’s impact.

Along with patient preferences and motivation, patient expectations are also a factor to consider. Pérez-Aranda et al. (2019b) addresses this topic, describing MBSR receiving higher ratings on treatment-expectations compared to FibroQoL, with significant group differences but with no association with treatment outcomes. Since placebo interventions were shown to have some efficacy in fibromyalgia (Chen et al., 2017), we hypothesize that fibromyalgia patients’ preferences, expectations and motivation for MBI programs integration are enhancers to its beneficial effects. It was recently demonstrated that expectancies and believes of participants play a stronger role in attenuating acute pain in novices following brief mindfulness interventions than the actual mindfulness-specific processes or instructions delivered (Davies et al., 2022). Future research in this promising topic may help develop more tailored indications for MBI treatments in fibromyalgia.

As referred above, the seven analyzed studies used a large diversity of outcomes, most of which were only based in self-reports of the participants. The discovery of biomarkers of the disease may allow to more objectively understand how MBI acts in fibromyalgia. The investigation of the mechanisms of fibromyalgia is expected to provide important results in the next years. In fact, as to the neurobiological mechanisms referred in the Introduction, namely the immune-inflammatory connection, recent studies indicated that MBSR has regulatory effects of the immune response since it partially corrected the imbalance in the ration of pro- and anti-inflammatory cytokines, along with Brain-Derived Neurotrophic Factor (BDNF) (Andres-Rodriguez et al., 2019; Sanabria-Mazo et al., 2020). As to the “brain hypothesis,” and considering that impaired endogenous pain modulation was detected by imaging studies in fibromyalgia patients (Staud, 2011), an interesting study showed that the regional cerebral blood flow is altered in patients with fibromyalgia subjected to mindfulness interventions (Medina et al., 2022). Although we consider that objective biomarkers are necessary for research in MBI for fibromyalgia, we expected that they will be evaluated along with self-reports. Approaches based in cost-utility evaluations of the effects of MBI in fibromyalgia which also include self-reports have shown to be useful (Pérez-Aranda et al., 2019a) and in what concerns pain it is “always a personal experience that is influenced to varying degrees by biological, psychological, and social factors” and “a person’s report of an experience as pain should be respected” (Raja et al., 2020).

In conclusion, research in mindfulness for fibromyalgia needs to be optimized to provide more grounded results (Davidson and Kaszniak, 2015; Van Dam et al., 2018). The specificities of fibromyalgia, the challenges in disease detection and the lack of biomarkers, add to the limitations of mindfulness interventions’ research for these patients. Research in mindfulness effects for fibromyalgia should continue taking into account the conceptual and methodological specificities inherent to research in mindfulness interventions itself.

## Author Contributions

Both authors have contributed equally to the research, design, analysis, and writing and approved the submitted version.

## Conflict of Interest

The authors declare that the research was conducted in the absence of any commercial or financial relationships that could be construed as a potential conflict of interest.

## Publisher’s Note

All claims expressed in this article are solely those of the authors and do not necessarily represent those of their affiliated organizations, or those of the publisher, the editors and the reviewers. Any product that may be evaluated in this article, or claim that may be made by its manufacturer, is not guaranteed or endorsed by the publisher.
